# Detection of Abnormal Events via Optical Flow Feature Analysis

**DOI:** 10.3390/s150407156

**Published:** 2015-03-24

**Authors:** Tian Wang, Hichem Snoussi

**Affiliations:** 1 School of Automation Science and Electrical Engineering, Beihang University, Beijing 100191, China; 2 Institut Charles Delaunay-LM2S-UMR STMR 6279 CNRS, University of Technology of Troyes, Troyes 10004, France; E-Mail: hichem.snoussi@utt.fr

**Keywords:** abnormal detection, optical flow, one-class SVM, KPCA

## Abstract

In this paper, a novel algorithm is proposed to detect abnormal events in video streams. The algorithm is based on the histogram of the optical flow orientation descriptor and the classification method. The details of the histogram of the optical flow orientation descriptor are illustrated for describing movement information of the global video frame or foreground frame. By combining one-class support vector machine and kernel principal component analysis methods, the abnormal events in the current frame can be detected after a learning period characterizing normal behaviors. The difference abnormal detection results are analyzed and explained. The proposed detection method is tested on benchmark datasets, then the experimental results show the effectiveness of the algorithm.

## 1. Introduction

With the development of human society, the security challenges in public scenes are gradually increased. In the last several decades, the cost for camera and network communication has been significantly reduced. Furthermore, video camera sensors are used wildly in many areas of human life. However, the traditional way of visual surveillance is a labor-intensive non-stop human attention work, and the efficiency is low. Thus, tackling visual surveillance problems automatically by adopting video processing technique plays a paramount role in the computer vision research area. The scientific challenges in this area include developing strategies to ensure public safety and detecting the abnormal behavior of an individual or a group.

The methods modeling behavior by adopting the Bayesian network were introduced in [1–4]. In [5], delta-dual hierarchical Dirichlet processes (dDHDP) were used to detect abnormal activity patterns in the field of visual features. By analyzing the statistical property, abnormal events were detected. Successful results were obtained on several scenes, but the prediction was based on the complicated probability model.

Some researchers took notice of spatio-temporal features. In [6], the movement was represented by a co-occurrence matrix and modeled by a Markov random field model. Abnormal activities, which were the significant changes in the scene, were detected. The work was similar to the foreground subtraction method in a non-stable background scene.

Low-level motion features have also gained attention for detecting abnormal events. In [7], an algorithm was proposed to detect the action of a single individual, such as hand-waving, boxing, *etc*. In [8], bionics technology was applied to model the superior colliculus (SC) to discover abnormalities in the panoramic image. These methods were based on partial information, such as contained in small observation windows of the image. In other words, they did not employ global information within the frame.

**Figure 1:**
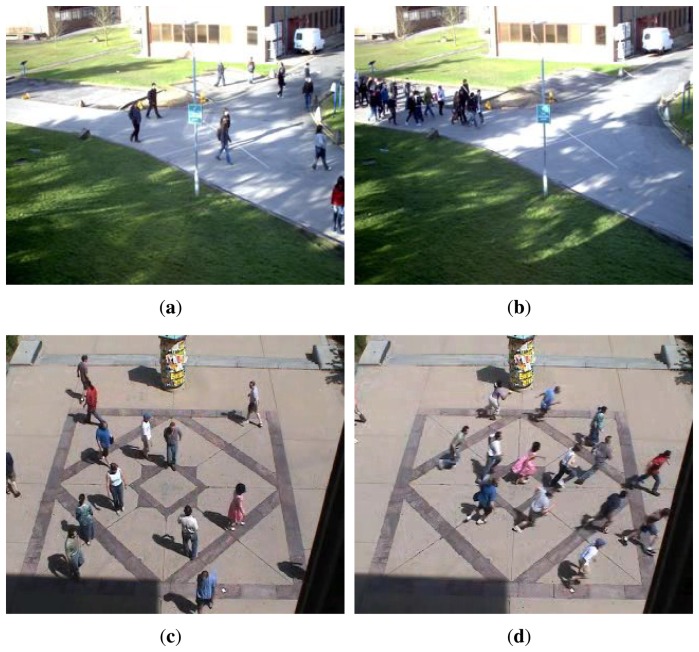
Normal and abnormal frames: (**a**,**c**) normal frames in the PETS and UMN datasets; the individuals are walking in all directions; (**b**) PETS abnormal frame; the individuals are walking in the same direction; (**d**) UMN abnormal frame; the individuals are running in all directions.

Based on the feature representation and the pattern classification, an abnormal detection method is proposed in this paper. The datasets used in our work are Performance Evaluation of Tracking and Surveillance (PETS2009) [9] and University of Minnesota (UMN) dataset [10], as shown in Figure 1. A normal scene means that the individuals are promenading in different directions. In the abnormal scenes of the PETS dataset, people are moving (walking or running) in the same direction, while the UMN abnormal scene means that the individuals are running. The proposed algorithm is composed of two parts. Firstly, the visual features are extracted without object tracking. Secondly, abnormal events are detected by classifying the extracted features. In fact, one-class support vector machine (SVM) and principal component analysis (PCA) are used in this paper. By learning the normal behaviors, the classifiers detect the abnormal ones. The rest of the paper is organized as follows. In Section 2, the optical flow-based feature is proposed. In Section 3, a one-class SVM classification method and a kernel PCA for novelty detection method are presented, and thus, the corresponding abnormal detection framework is described. In Section 4, the experimental results and the analysis are given. Finally, the paper is concluded with future works in Section 5.

## 2. Feature Selection for Abnormal Detection

Because optical flow can represent the movement information of actions, we choose the Horn-Schunck (HS) [11] method to compute it. The HS method formulates the optical flow as a global energy functional for the gray image sequence:

(1) $$E=\int \int \left[{\left(I_{x}u+I_{y}v+I_{t}\right)}^{2}+\alpha \left({\left\|\nabla u\right\|}^{2}+{\left\|\nabla v\right\|}^{2}\right)\right]dxdy$$

where *I_x_*,*I_y_* and *I_t_* are the derivatives of the image intensity values along the horizontal direction *x*, vertical direction *y* and time *t* dimension, respectively, *u*,*v* are the horizontal and vertical components of the optical flow, *α* is a regularization constant.

**Figure 2:**
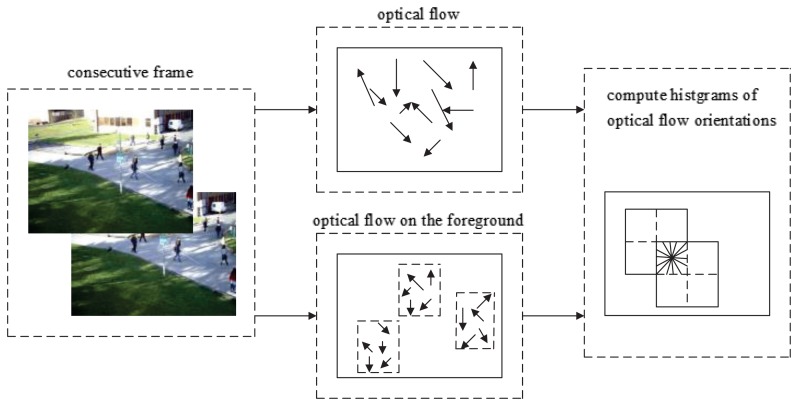
Histogram of the optical flow orientation (HOFO) feature descriptor on the original image or the foreground image.

In [12], the abnormal global frame detection was proposed, and the frame covariance matrix descriptor was constructed based on the optical flow. In this paper, we analyze the details of the histogram of the optical flow orientation (HOFO) with different parameters. The optical flow orientation features of an image are extracted at fixed resolution and then gathered into a high dimensional feature vector. A 2 × 2 rectangular cell HOFO descriptor of the original image or the foreground image is shown in Figure 2. By a trigonometric function, the orientation is computed from horizontal and vertical optical flow. The orientation is voted into *n* bins in 0°–360° (noted as *signed* angle) or 0°–180° (noted as *unsigned* angle). Nine bins are chosen in this paper. The optical flow magnitude of a pixel is considered as a weight coefficient in the voting process. A block contains *h_b_* × *w_b_* cells; it is set as 2 × 2 in this paper to present the spatial information of the HOFO. The HOFO dimension of one block is 36 (9×2×2). The HOFO feature describes the global movement information of one frame (or foreground frame) by gathering the histogram of the optical flow orientation in the sub-frame (block). Because the movement of an abnormal image usually has a bigger value of the optical flow strength and more directions, the element in the HOFO vector of an abnormal image is generally higher than a normal one. Four normalization schemes are chosen when HOFO is calculated:

(2) $$L1-\text{norm}:\text{v}\;\to \frac{\text{v}}{{\left\|\text{v}\right\|}_{1}+\varepsilon }$$

(3) $$L1-\mathrm{sqrt}:\text{v}\;\to \sqrt{\frac{\text{v}}{{\left\|\text{v}\right\|}_{1}+\varepsilon }}$$

(4) $$L2-\text{norm}:\text{v}\;\to \sqrt{\frac{\text{v}}{{\left\|\text{v}\right\|}_{2}^{2}+\varepsilon }}$$

(5) $$L2-\text{Hys}:\text{v}\;\to \sqrt{\frac{\text{v}}{{\left\|\text{v}\right\|}_{2}^{2}+\varepsilon }}$$

(6) $$\{\begin{array}{cc} if & {\left\|\text{v}\right\|}_{2}^{2}>0.3,{\left\|\text{v}\right\|}_{2}^{2}=0.3\\ if & {\left\|\text{v}\right\|}_{2}^{2}>0.4,{\left\|\text{v}\right\|}_{2}^{2}=0.4 \end{array}$$

where *v* is the HOFO descriptor vector before being normalized and *ε* is a small constant to make the calculation reasonable.

## 3. Abnormal Detection Method Based on Optical Flow Analysis

The objective of the abnormal event detection problem is to find the samples that are different from the training ones. Thus, two classification methods, the one-class support vector machine (one-class SVM) and kernel principal component analysis (KPCA) for novelty detection, suit this application. In this section, we firstly introduce these two methods and then propose the abnormal detection algorithm in video sequences.

### 3.1. One-Class Support Vector Machine

Vapnik and Lerner initially proposed the support vector machine for classification or regression based on statistical learning theory [13]. Later, by adopting the kernel methods, the support vector machine was extended to deal with non-linear problems [14–16]. Thus, the non-linear one-class support vector machine is one development of the basic SVM theory to find out an appropriate region containing most of the data drawn from an unknown probability distribution. The problem of the non-linear one-class support vector machine can be presented as [17,18]:

(7) $$\begin{array}{ll} \underset{\omega ,\xi ,\rho }{\min}\frac{1}{2}{\left\|w\right\|}^{2}+\frac{1}{\nu n}\overset{n}{\underset{i=1}{\text{∑}}}\xi_{i}-\rho & \\ \text{subject to}\;\langle w,\Phi \left(x_{i}\right)\rangle \geq \rho -\xi_{i}, & \xi_{i}\geq 0 \end{array}$$

where *x_i_* ∈ *χ*, *i* ∈ [1 … *n*] are *n* training samples in the original data space *χ*. ξ*_i_* is the slack variable for penalizing the outliers. The hyperparameter *ν* ∈ (0,1; is the weight for the controlled slack variable. Φ is a map from the non-empty set of the original input data *χ* to a feature space 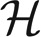. For computing dot products in 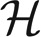, the kernel function is defined as *κ*(***x***,***x′***) = 〈Φ(***x***) · Φ(***x′***)〉. The decision function is defined as:

(8) $$f\left(x\right)=\mathrm{sgn}\left(\sum_{i=1}^{n}\alpha_{i}\kappa \left(x_{i},x\right)-\rho \right)$$

where ***x*** is a vector in the input data space *χ* and *κ* is the kernel function. The Gaussian kernel is used to deal with the non-linear problem in this paper.

### 3.2. Kernel Principal Component Analysis

Kernel principal component analysis [19,20] extends the standard PC A to non-linear data distributions. Before performing PCA, map the *n* datum points **x***_i_* ∈ ℝ*^d^* to a higher-dimensional feature space 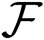 where standard PCA is performed:

(9) $$x_{i}\to \Phi \;\left(x_{i}\right)$$

In kernel PCA, an eigenvector *V* of the covariance matrix in 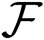 is a linear combination of Φ(***x**_i_*):

(10) $$V=\sum_{i=1}^{n}\alpha_{i}\tilde{\Phi }\left(x_{i}\right)$$

(11) $$\tilde{\Phi }\left(x_{i}\right)=\Phi \left(x_{i}\right)-\frac{1}{n}\sum_{r=1}^{n}\Phi \left(x_{r}\right)$$

where *α_i_* is the component of a vector ***α***. This vector is an eigenvector of the matrix *k̃* (***x**_i_*,***x**_j_*) = 〈 (Φ̃(***x**_i_*) · Φ̃(***x**_j_*)〉.

For novelty detection [21], the reconstruction error *p*(Φ̃); can be defined as:

(12) $$p\left(x\right)=p_{S}\left(x\right)-\sum_{l=1}^{q}f_{l}{\left(x\right)}^{2}$$

subject to:

(13) $$p_{S}\left(x\right)={\left\|\Phi \left(x\right)-\frac{1}{n}\sum_{r=1}^{n}\Phi \left(x_{r}\right)\right\|}^{2}$$

$$\begin{array}{ll} f_{l}\left(x\right) & =\langle \tilde{\Phi }\left(x\right)\cdot V^{l}\rangle \\ & =\overset{n}{\underset{i=1}{\text{∑}}}\alpha_{i}^{l}[k\left(x,x_{i}\right)-\frac{1}{n}\overset{n}{\underset{r=1}{\text{∑}}}k\left(x_{i},x_{r}\right)-\\ & \frac{1}{n}\overset{n}{\underset{r=1}{\text{∑}}}k\left(x,x_{r}\right)+\frac{1}{n^{2}}\overset{n}{\underset{r,s=1}{\text{∑}}}k\left(x_{r},x_{s}\right)] \end{array}$$

where *f_l_*(***x***) is the projection of **Φ̃**(***x***) on the eigenvector ***V**^l^*, and the index *l* denotes the *l*-th eigenvector, with *l* = 1 for the eigenvector with the largest eigenvalue.

### 3.3. Abnormal Detection Algorithm Based on Optical Flow Feature Classification

By adopting the histogram of the optical flow orientation feature descriptor and these two novel detection methods, the abnormal event detection method in video streams is summarized in Algorithm 1. [*I*_1_ … *I_m_*] is the set of normal training frames where the individuals are walking in all directions. The abnormal samples are the frames where the individuals are moving toward one direction or running. This definition of an abnormal event indicates that the individuals are attracted by some particular event or escaping from a dangerous zone.



**Algorithm 1** Abnormal detection algorithm.
**Require:**Image *I*.1:Compute the optical flow of training frame [*I*_1_,…, *I_m_*] via the HS method.
$\left[I_{1},I_{2},\ldots ,I_{m}\right]\to \left[O_{1},O_{2},\ldots ,O_{m}\right]$2:Compute the histogram of the optical flow orientation of the original image or foreground image.
$$\left[O_{1},O_{2},\ldots ,O_{m}\right]\to \left[H_{1},\ldots ,H_{m}\right]$$3:
(1)SVM method: training data are learned by the one-class SVM method to obtain the support vectors.
$\left[H_{1},\ldots ,H_{m}\right]\;\underset{\to }{\text{SVM}}\;\text{support vector}\;[S_{1},\ldots ,S_{m_{1}}]$(2)PCA method: compute the principal components by the KPCA method and measure the squared distance.
$$\left[H_{1},\ldots ,H_{m}\right]\;\underset{\to }{\text{PCA}}\;\text{principal component}\;[P_{1},\ldots ,P_{m_{2}}]$$4:
(1)SVM method: Each incoming frame *H_n_*_,…,_*_q_* is classified by one-class SVM. The abnormal event or normal event is detected in the current image.(2)PCA method: each incoming frame *H_n_*_,…,_*_q_* is classified by KPCA.5:The detection results are filtered by state transition restriction.

**Figure 3:**
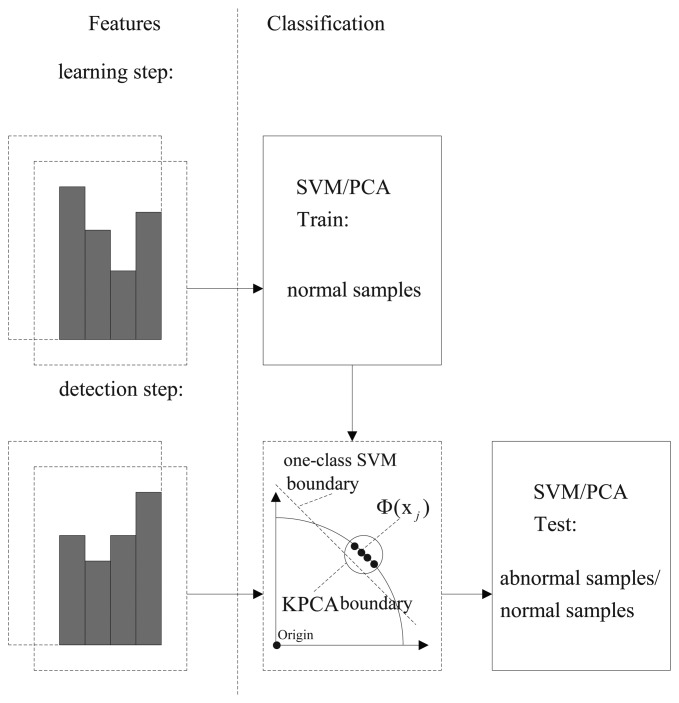
The flowchart of the proposed feature classification-based abnormal detection method.

Step 1 Compute the optical flow of each frame via the Horn–Schunck (HS) optical flow method in the gray scale.
Step 2 Calculate the histogram of the optical flow orientation (HOFO) of each frame. The sketch image for choosing the HOFO feature in the original image or in the foreground image is shown in Figure 2. If the HOFO descriptor is computed on the foreground image, the optical flow in the background is zero. Thus, the background area is not considered, and then, computing time is saved.
Step 3 The one-class support vector machine or kernel principal component analysis method is used to classify feature samples of the incoming video frames. The flowchart of our method is shown in Figure 3.
  SVM method The training feature samples are extracted from the normal images, which include HOFO in the original images or in the foreground images. The HOFO feature of the *k*-th frame is labeled as *H_k_*. The training samples *H*_1,…,_*_m_*, *m* > 1 are gathered, and then, the support vectors are obtained in the SVM training step. Based on the support vectors, the incoming feature samples *H_n_*_,…,_*_q_* are classified.
  PCA method The normal training feature samples for KPCA are mapped into a high-dimensional feature space. In this space, PCA extracts the principal components of the data distribution. Then, the squared distance of each testing sample to the corresponding principal subspace is measured for novelty detection [21].
Step 4 If a normal event or an abnormal event is observed, it means that the video clip holds one state in several consecutive frames. Thus, we use a state transition restriction method by presetting a threshold *N* to filter the short fluctuation clip. If the number of the predicted abnormal frames after a normal video clip is larger than *N*, the state of the abnormal detection system is changed from “normal” to “abnormal”.

## 4. Experimental Results and Analyses

This section presents the experimental results and the analyses of the proposed abnormal detection method. The datasets PETS [9] and UMN [10] are used.

### 4.1. PETS Dataset

**Figure 4:**
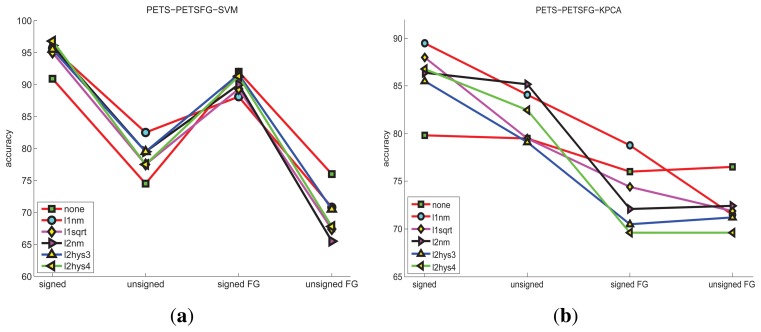
The accuracy of the PETS scene under different classification methods and different features: (**a**) HOFO descriptors are computed on the *original* and the *foreground* image, and one-class SVM is taken as the classification method, the maximum accuracy is 96.8% ; (**b**) HOFO descriptors are computed on the *original* image and the *foreground* image, and KPCA is taken as the classification method, the maximum accuracy is 89.5%.

**Figure 5:**
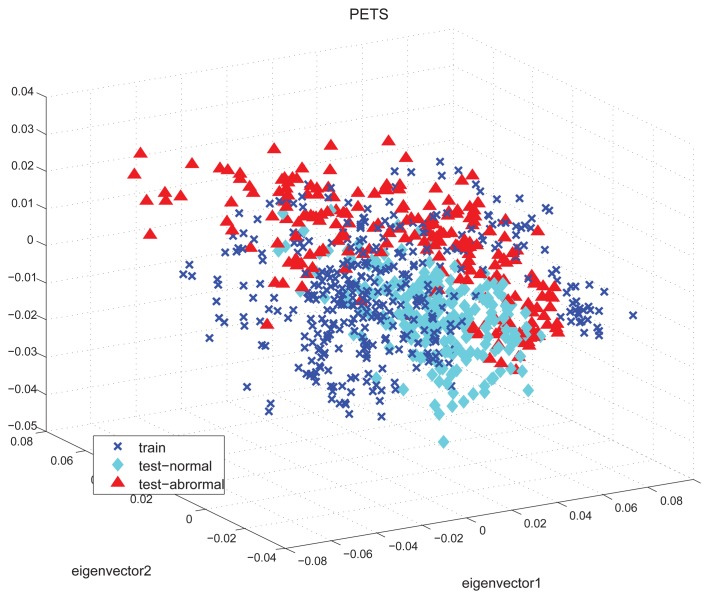
The normal data for training, normal data for testing and abnormal data for testing of the three largest principal components in the PETS scene.

The detection accuracies of PETS dataset under different features and classification methods are shown in Figure 4. The HOFO features are obtained under different conditions, which include *original* image, *foreground* image, *signed* angle, *unsigned* angle, *L*1-*norm*, *L*1-*sqrt, L*2-*norm*, *L*2*hys*-0.3, *L*2*hys*-0.4 and *none* normalization. The KPCA novelty detection method obtains less accuracy than the one-class SVM. The best accuracy of the KPCA results is 89.5% under the condition of the *original* image, *signed* angle and *L*1-*norm* normalization. The best accuracy of the one-class SVM is 96.8% under the condition of the *original* image, *singed* angle and *L*2*hys*-0.4 normalization. Examples of this high dimension feature space are illustrated in Figure 5 by using the projection of the three largest principal components. The training normal data (labeled as a blue cross) are confused with the testing normal data (labeled as a cyan diamond) and the testing abnormal data (labeled as a red rectangle). In a word, the training data are mixed with the test-abnormal data. One-class SVM has a slack variable, which tunes the number of acceptable outliers of the training data. This soft margin strategy makes the one-class SVM obtain more precision.

**Figure 6:**
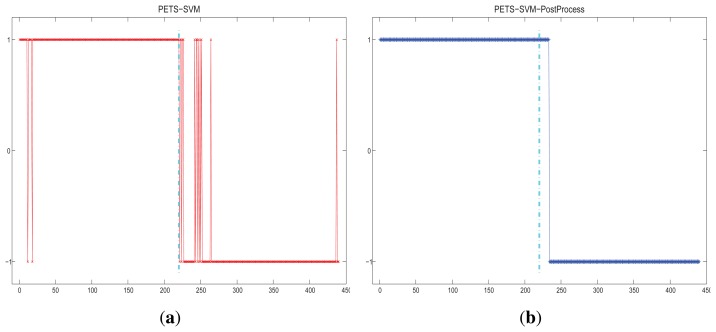
Detection results of the PETS scene. “1” means normal; “–1” means abnormal: (**a**) original detection results; (**b**) detection results that are post-processed by the state transition restriction. The consecutive frame number threshold *N* = 8.

**Figure 7:**
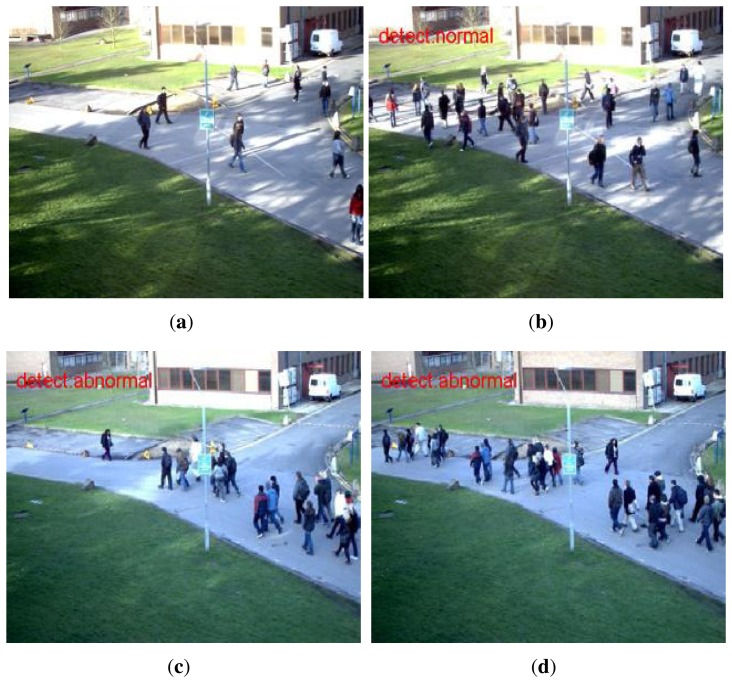
Detection results of the PETS scene: (**a**) training frames; people are walking in all directions; 130 frames are used for training; (**b**) a normal frame; (**c**,**d**) abnormal frames; people are walking in the same direction. Two hundred and nineteen frames are used for normal and abnormal testing, respectively.

The results adjusted by restriction of the state transition are shown in Figure 6. As shown in the figure, the fluctuations between the “abnormal” and “normal” state are reduced. The detection results of the PETS scene are shown in Figure 7.

### 4.2. UMN Dataset

**Figure 8:**
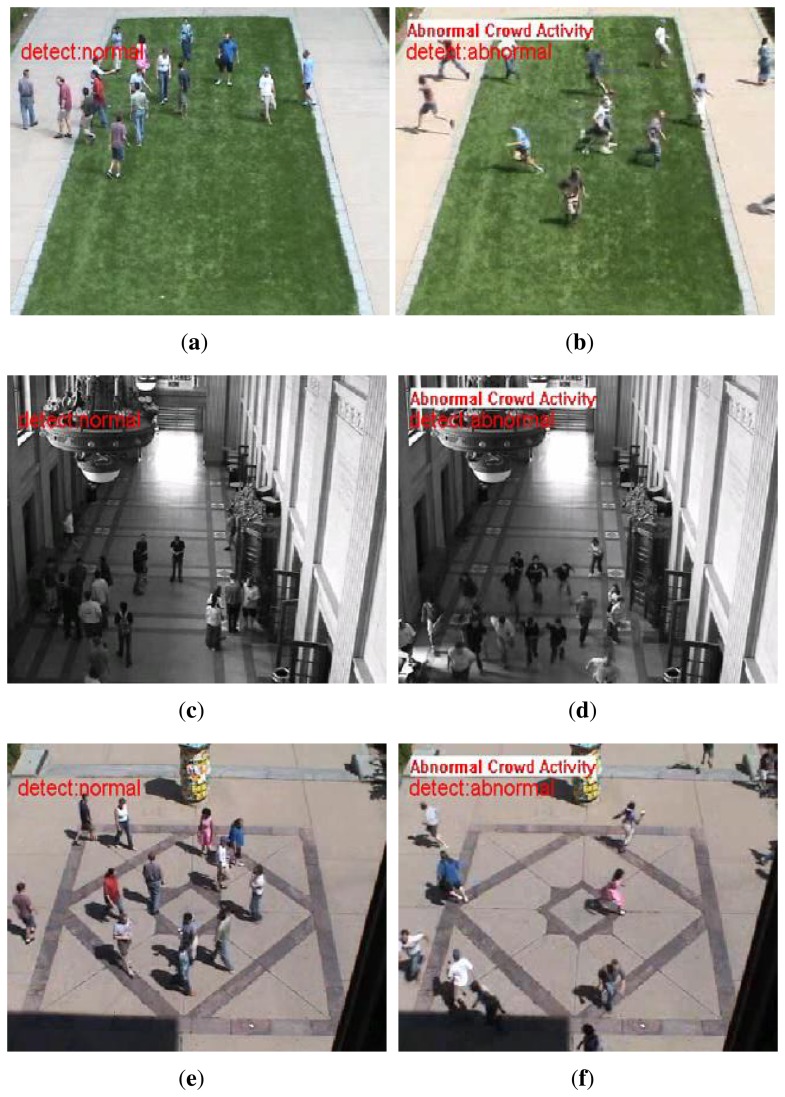
Detection results of the UMN scenes. Normal events are those where the individuals are walking; abnormal events are those where the individuals are running: (**a**,**c**,**e**) normal frames of lawn, indoor and plaza scene; (**b**,**d**,**f**) abnormal frames of lawn, indoor and plaza scene.

The HOFO descriptor can represent not only the information of optical flow orientation, but also the optical flow magnitude. The results of the benchmark dataset UMN are shown in Figure 8. The HOFO descriptor can deal with the abnormal scene in which people are running in all directions.

**Figure 9:**
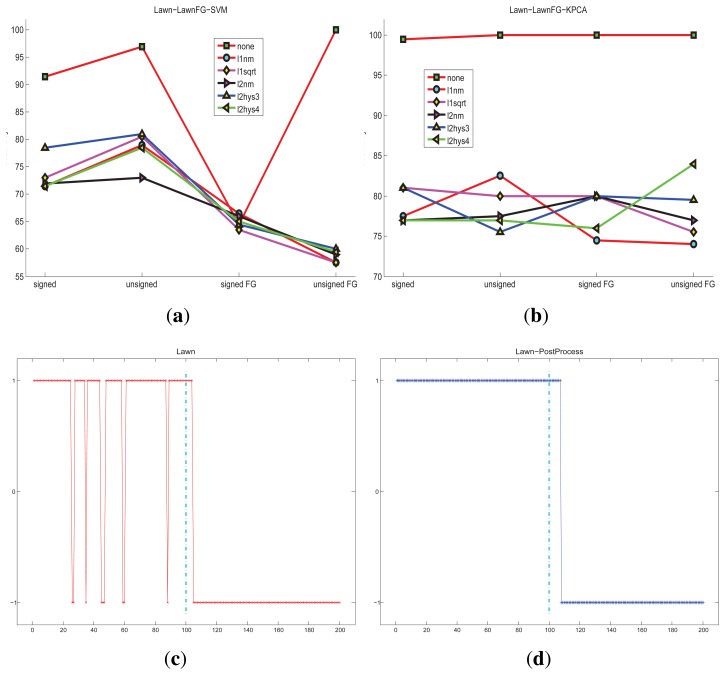
The accuracy of the lawn scene under different classification methods and different features: (**a**) HOFO descriptors are computed on the *original* image and the *foreground* image; one-class SVM is taken as the classification method, the maximum accuracy is 100%; (**b**) HOFO descriptors are computed on the *original* image and the *foreground* image; KPCA is taken as the classification method; (**c**) original detection results, the maximum accuracy is 100%; (**d**) detection results that are post-processed by the state transition restriction strategy; the consecutive frame number threshold is *N* = 3. Four hundred and eighty normal frames are used for training; 100 frames are used for normal and abnormal testing, respectively.

For the lawn scene, the detection accuracies of different conditions are shown in Figure 9. One-class SVM and KPCA classification methods can get great accuracy without the state transition restriction strategy.

**Figure 10:**
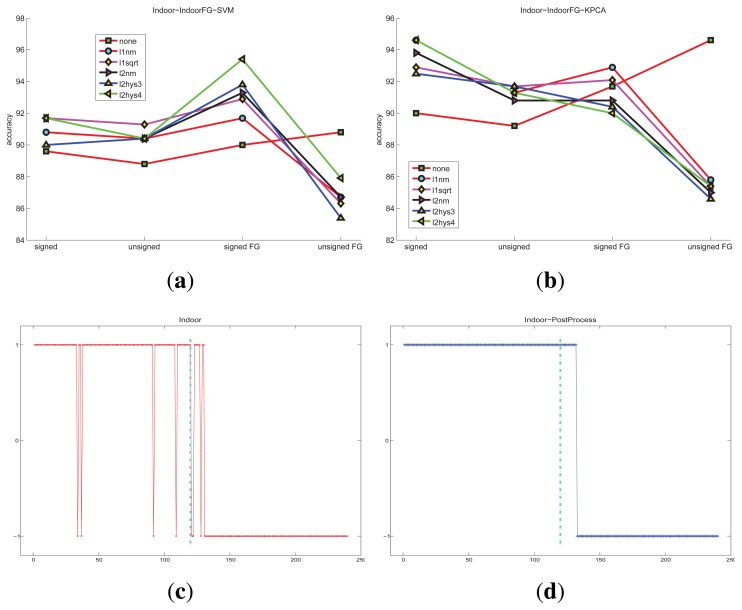
The accuracy of the indoor scene under different classification methods and different features: (**a**) HOFO descriptors are computed on the original image and the foreground image; one-class SVM is taken as the classification method, the maximum accuracy is 95.4%; (**b**) HOFO descriptors are computed on the original image and the foreground image; KPCA is taken as the classification method; (**c**) original detection results, the maximum accuracy is 94.6%; (**d**) detection results that are post-processed by the state transition restriction strategy; the consecutive frame number threshold is *N* = 3. 250 frames are used for training; 120 frames are used for normal and abnormal testing, respectively.

**Figure 11:**
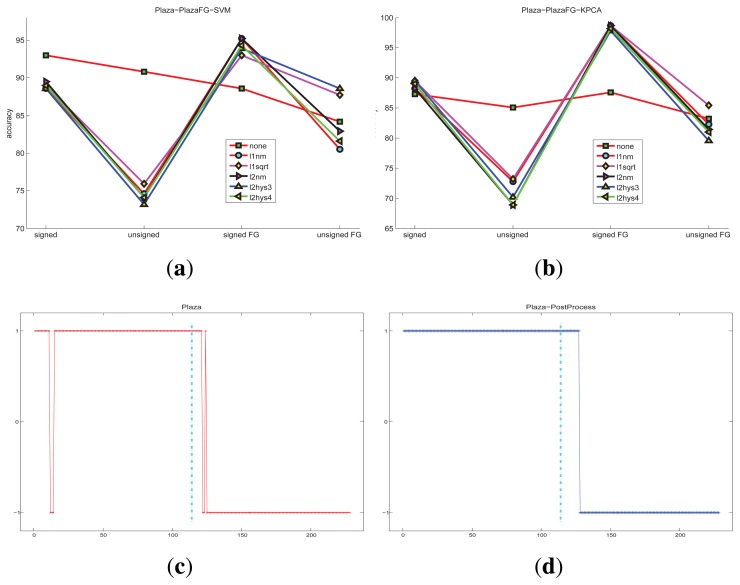
The accuracy of the plaza scene under different classification methods and different features: (**a**) HOFO of the original image and the foreground image; one-class SVM is taken as the classification method, the maximum accuracy is 95.2%; (**b**) HOFO of the original image and the foreground image; KPCA is taken as the classification method, the maximum accuracy is 98.7%; (**c**) original detection results; (**d**) detection results that are post-processed by the state transition restriction strategy; the consecutive frame number threshold is *N* = 4. 250 normal frames are used for training; 114 frames are used for normal and abnormal testing, respectively.

For the indoor scene and the plaza scene, the detected accuracy of different conditions are shown in Figures 10 and 11, respectively The restriction of the state transition improves the accuracy In summary, the KPCA method is generally better than one-class SVM for abnormal detection in these experiments. Furthermore, the data distributions need to be considered.

The performance summary of the UMN dataset compared with the state-of-the-art methods is shown in Table 1. The results in the table are not post-processed by the state transition restriction strategy. Our method obtains great accuracy for all three scenes in the UMN dataset.

## 5. Conclusions

We propose an abnormal detection method by analyzing the optical flow feature. The method is based on two components, computing the histogram of the optical flow orientation (HOFO) and applying one-class support vector machine and kernel principal component analysis for classification. The HOFO feature is computed in the original frame or foreground image. Moreover, the details of the parameters are analyzed. The algorithm has been tested on several video sequences, and the experimental results show the effectiveness of the algorithm. From the experimental results, we can see that the normalization schemes, *none* and *L*2*hys*-0.4, generally get the best performance. The detection results under the *signed* angle and *original* image condition is broadly acceptable. In general, the KPCA novelty detection method is as good as one-class SVM, but under a certain distribution of the data, the one-class SVM can obtain more accurate performance.

Future work will aim at reducing the false alarms and training the samples online. Two solutions are under consideration: capturing more efficient features based on the optical flow or replacing the optical flow by other approaches that can represent the information of the events. Online learning is also urgent. Due to the large amount of normal examples, it is hard to learn the training samples as one batch. Moreover, our method focuses on detecting global abnormal events, but detecting local abnormal events is also important. Improving the method to detect the global and local abnormal events jointly is also necessary.

## Figures and Tables

**Table 1:** The comparison of our proposed method with the state-of-the-art methods for global abnormal event detection in the UMN dataset. TPR, true positive rate; FPR, false positive rate. NN, nearest neighbor. SRC, sparse reconstruction cost. STCOG, spatial-temporal co-occurrence Gaussian mixture models.

**Method**	**Area under ROC**		

	**Lawn**	**Indoor**	**Plaza**
Social Force [22]		0.96	
Optical Flow [22]		0.84	
NN [23]		0.93	
SRC [23]	0.995	0.975	0.964
STCOG [24]	0.9362	0.7759	0.9661
HOFO SVM (Ours)	0.9845	0.9037	0.9815
HOFO PCA (Ours)	0.9992	0.9880	0.9989

## References

[1] Utasi Á., Czúni L. (2010). Detection of unusual optical flow patterns by multilevel hidden markov models. Opt. Eng..

[2] Kosmopoulos D., Chatzis S.P. (2010). Robust visual behavior recognition. IEEE Signal Process. Mag..

[3] Xiang T., Gong S. (2008). Incremental and adaptive abnormal behaviour detection. Comput. Vis. Image Underst..

[4] Jiménez-Hernández H., González-Barbosa J.J., Garcia-Ramírez T. (2010). Detecting abnormal vehicular dynamics at intersections based on an unsupervised learning approach and a stochastic model. Sensors.

[5] Haines T.S., Xiang T. Delta-dual hierarchical dirichlet processes: A pragmatic abnormal behaviour detector.

[6] Benezeth Y., Jodoin P.M., Saligrama V. (2011). Abnormality detection using low-level co-occurring events. Pattern Recog. Lett..

[7] Schuldt C., Laptev I., Caputo B. Recognizing human actions: A local svm approach.

[8] Casey M.C., Hickman D.L., Pavlou A., Sadler J.R. Small-scale anomaly detection in panoramic imaging using neural models of low-level vision.

[9] PETS 2009 Benchmark Data. Multisensor Sequences Containing Different Crowd Activities. http://www.cvg.rdg.ac.Uk/PETS2009/a.html.

[10] Unusual Crowd Activity Dataset of University of Minnesota. http://mha.cs.umn.edu/Movies/Crowd-Activity-All.avi.

[11] Horn B.K., Schunck B.G. (1981). Determining optical flow. Artif. Intell..

[12] Wang T., Chen J., Snoussi H. (2013). Online detection of abnormal events in video streams. J. Electr. Cmoput. Eng..

[13] Vapnik V.N., Lerner A. (1963). Pattern recognition using generalized portrait method. Autom. Remote Control.

[14] Boser B.E., Guyon I.M., Vapnik V.N. A training algorithm for optimal margin classifiers.

[15] Piciarelli C., Micheloni C., Foresti G.L. (2008). Trajectory-based anomalous event detection. IEEE Trans. Circuits Syst. Video Technol..

[16] Cristianini N., Shawe-Taylor J. (2000). An Introduction to Support Vector Machines and Other Kernel-Based Learning Methods.

[17] Schölkopf B., Platt J.C., Shawe-Taylor J., Smola A.J., Williamson R.C. (2001). Estimating the support of a high-dimensional distribution. Neural Comput..

[18] Canu S., Grandvalet Y., Guigue V., Rakotomamonjy A. (2005). Svm and Kernel Methods Matlab Toolbox.

[19] Schölkopf B., Smola AJ. (2002). Learning with Kernels: Support Vector Machines, Regularization, Optimization and Beyond.

[20] Schölkopf B., Smola A., Müller K.R. (1998). Nonlinear component analysis as a kernel eigenvalue problem. Neural Comput..

[21] Hoffmann H. (2007). Kernel PCA for novelty detection. Pattern Recog..

[22] Mehran R., Oyama A., Shah M. Abnormal crowd behavior detection using social force model.

[23] Cong Y., Yuan J., Liu J. Sparse reconstruction cost for abnormal event detection.

[24] Shi Y., Gao Y., Wang R. Real-time abnormal event detection in complicated scenes.

